# Human stem cells express pannexins

**DOI:** 10.1186/s13104-018-3125-z

**Published:** 2018-01-22

**Authors:** Nadine Hainz, Anja Beckmann, Madline Schubert, Alexandra Haase, Ulrich Martin, Thomas Tschernig, Carola Meier

**Affiliations:** 1 Department of Anatomy and Cell Biology, Saarland University, Kirrberger Straße, Building 61, Saar, 66421 Homburg, Germany; 2 Leibniz Research Laboratories for Biotechnology and Artificial Organs (LEBAO), Department of Cardiac, Thoracic, Transplant and Vascular Surgery, Hannover Medical School, 30625 Hannover, Germany; 3 REBIRTH-Cluster of Excellence, Hannover Medical School, 30625 Hannover, Germany

**Keywords:** Pannexins, Human stem cells, Differentiation, Endoderm, Regulation

## Abstract

**Objective:**

Pannexins are channel proteins important for the release of calcium and adenosine triphosphate, which are among other functions involved in early development. Here, the expression of pannexins was investigated in induced pluripotent stem cells derived from human cord blood endothelial cells (hCBiPS2), in hematopoietic stem cell-derived induced pluripotent stem cells (HSC_F1285_T-iPS2) and in human embryonic stem cells (HES-3). The expression of pannexin (Panx) 1–3 mRNAs was analyzed in all three undifferentiated stem cell lines. Stem cells then underwent undirected differentiation into embryoid bodies and were analyzed regarding expression of germ layer-specific genes.

**Results:**

Panx1, Panx2, and Panx3 mRNAs were expressed in all undifferentiated stem cell lines investigated. In comparison, Panx1 showed the highest expression among all pannexins. The undirected differentiation resulted in a mixed germ layer genotype in all three stem cell lines. Whereas the expression of Panx1 was not affected by differentiation, the expression of Panx2 was slightly increased in differentiated hCBiPS2 cells, HSC_F1285_T-iPS2 as well as HES3 cells as compared to their undifferentiated counterparts. A slight increase of Panx3 expression was observed in differentiated hCBiPS2 cells only. In conclusion, pluripotent stem cells express all three pannexin genes.

**Electronic supplementary material:**

The online version of this article (10.1186/s13104-018-3125-z) contains supplementary material, which is available to authorized users.

## Introduction

Although the pannexin family was discovered already in 2000, little is known on expression of its members in stem cells [1]. Pannexins are highly conserved proteins, which form transmembrane channels [2, 3]. These channels are involved in calcium release and ATP release [4]. Pannexins are functionally linked to adenosine receptors and activate the inflammasome after ATP stimulation. Pannexin (Panx) 1 is widely expressed in many organs including the brain. Panx2 was primarily detected in the brain [5]. Panx3, in contrast, is present in skin, bone and cartilage tissue but absent from the nervous system [6, 7]. Pannexins are involved in many physiological processes and play a role in many diseases or disease models [8–17]. They are associated with regulation of the cell cycle and induction of apoptosis and are expressed during early development of the central nervous system [18].

However, few data are available on the expression and function of pannexins in stem cells: Panx3 was found to inhibit the proliferation of osteo-progenitor cells via interaction with regulatory pathways [19]. In contrast, Panx1 supported the proliferation in neural stem and progenitor cells via the release of ATP [20–22]. Panx1 and Panx3 are both involved in the proliferation of skeletal muscle myoblast proliferation and differentiation [10]. As those studies demonstrate expression and function of pannexins in multipotent stem cells, their function in pluripotent stem cells is also feasible. In the presented investigation, expression was therefore analyzed in three different pluripotent stem cell lines. The aim of this investigation was to study the expression of all three pannexins in the two induced pluripotent stem cell lines (hCBiPS2 and HSC_F1285_T-iPS2) as well as in human embryonic stem cells (HES-3). For each cell type, expression in undifferentiated stem cells was compared to that of undirected differentiated stem cells to analyze differentiation-associated changes in the expression of pannexins.

## Main text

### Methods

The hiPSC lines were generated by lentiviral transduction of cord blood-derived endothelial cells (hCBiPS2) as previously described [23]. hCBiPS2 cells were cultured on irradiated mouse embryonic fibroblasts (MEFs) in knockout Dulbecco’s modified Eagle’s medium (DMEM) supplemented with 20% knockout serum replacement, 1 mM l-glutamine, 0.1 mM β-mercaptoethanol, 1% nonessential amino acid stock (all from life technologies, Darmstadt, Germany), and 10 ng/ml basic fibroblast growth factor (bFGF; supplied by the Institute for Technical Chemistry, Leibniz University, Hannover, Germany) [24]. The human embryonic stem cell line HES-3 was cultured and expanded under standard hESC culture conditions. For EB-based differentiation, human pluripotent stem cells were detached from the feeder layer by collagenase IV, dispersed into small clumps and cultured in Iscove’s modified Dulbecco’s differentiation medium supplemented with 20% fetal calf serum, 1 mM l-glutamine, 0.1 mM beta-mercaptoethanol and 1% nonessential amino acid stock in ultra-low attachment plates (Corning) for 7 days. Subsequently, EBs were plated onto 0.1% gelatin-coated tissue culture dishes and cultivated for 11 days before RNA isolation.

Total RNA was isolated from cells using TRIzol Reagent (life technologies, Darmstadt, Germany) according to the manufacturer’s instructions. The RNA pellet was resuspended in RNase-free water. Afterwards, genomic DNA was eliminated by DNase digestion using RNAse-free DNase I set (Qiagen, Hilden, Germany, order number 79254). DNase I in RDD buffer was added to RNA solution and incubated for 10 min. After incubation, RNA solution was mixed with RLT buffer and 100% ethanol and was given onto an RNeasy Mini spin column (Qiagen, Hilden, Germany). After several washing steps, RNA was eluted from the RNeasy Mini spin column membrane with RNase-free water. Subsequently, the RNA was quantified using the spectrophotometer nano drop ND-1000 (Thermo Fisher Scientific, Heidelberg, Germany). Based on 500 ng RNA per sample, cDNA was synthesized using M-MuLV reverse transcriptase with oligo primer, dNTP-mix, RNase inhibitor and RT buffer (all reagents New England Biolabs, Frankfurt/Main, Germany). To exclude incomplete genomic DNA elimination, we performed controls without MuLV reverse transcriptase, and to test purity of used reagent/chemicals we performed a control without RNA. Polymerase chain reaction (PCR) was performed using 10× Thermo Polymerase Buffer, dNTP Mix, and Taq polymerase (all reagents New England Biolabs, Frankfurt/Main, Germany). For amplification of Panx1–3, specific forward and reverse primers were used (Additional file 1: Table S1). Primer design was performed with the NCBI primer-designing tool (http://www.ncbi.nlm.nih.gov/tools/primer-blast/; Panx1 primer sequences were taken from [3]). PCR products were separated by electrophoresis on a 2% agarose gel containing ethidium bromide for visualization of the PCR product. Quantitative real-time PCR (qPCR) was performed using the Takyon Rox SYBR MasterMix dTTP Blue (Eurogentec, Köln, Germany, order number UF-RSMT-B0701) according to the manufacturer’s instructions on a StepOnePlus Real-Time PCR System (life technologies, Darmstadt, Germany). In brief, cDNA was diluted 1:3 with water and then mixed with Takyon master mix and primers (Additional file 1: Table S2). All samples were measured in triplicate. The mean Ct of each triplet of the gene of interest was normalized to the mean Ct of the 18S rRNA gene as reference (ΔCt). The relative expression was calculated using the ΔΔCt method according to Pfaffl [25]. Thus, the mean Ct of the reference gene was subtracted from the mean Ct of the gene of interest (ΔCt). For Fig. 2, expression was then normalized to Panx1 (relative expression = 1; ΔΔCt); for Fig. 3, expression was normalized to the values obtained for undifferentiated cells. Relative mRNA expression was calculated as 2^−ΔΔCt^. Statistical analysis was performed by 2-way-ANOVA, followed by Tukey’s (for data in Fig. 2) or Sidak’s (for data in Fig. 3) multiple comparison test. p-values < 0.05 were considered statistically significant.

For immunoblotting analysis, proteins were isolated after RNA isolation with TRIzol Reagent (life technologies, Darmstadt, Germany) according to the manufacturer’s instructions. Samples were washed with 100% ethanol. After centrifugation, proteins were precipitated with isopropanol at 4 °C. After subsequent centrifugation, the pellets were washed with 0.3 M guanidine hydrochloride in ethanol and afterwards with 100% ethanol. For protein solubilization, pellets were resuspended in 1% SDS in water using a homogenizer (Precellys24, VWR, Darmstadt, Germany). 20 µg of protein extracts of stem cells were separated on a 10% SDS-PAGE. As positive controls, RIPA protein extracts of human cortex and human osteosarcoma SAOS-2 cells were used (see acknowledgements). After immunoblotting onto a polyvinylidene fluoride membrane, unspecific binding sites were blocked in Roti-Block blocking buffer (Roth, Karlsruhe, Germany) at room temperature for 30 min. Incubation with primary antibodies was performed at 4 °C overnight. Primary antibodies mouse Panx1 (R&D Systems, Minneapolis, MN, USA, order number MAB7097), rabbit Panx2 (AVIVA Systems Biology, San Diego, CA, USA, order number ARP42778_T100), rabbit Panx3 (Invitrogen, Camarillo, CA, USA, order number 433270), rabbit connexin 43 (abcam, Cambridge, UK, order number ab11370) and mouse actin (Sigma-Aldrich, Munich, Germany, order number A2103) were diluted in Roti-Block blocking buffer at dilutions of 1:500 (Panx1, Panx2, Panx3, actin) and 1:1000 (Cx43). After washing in TBST, secondary antibody incubation was performed at room temperature for 90 min. Secondary antibodies HRP-conjugated goat anti-mouse (Santa Cruz, Heidelberg, Germany, order number sc-2005) and goat anti-rabbit (Thermo Scientific, Schwerte, Germany, order number A10547) were diluted 1:7000 in Roti-Block blocking buffer. Development of signals was performed by incubation with ECl substrate (WesternBright Quantum, Menlo Park, CA; USA) for 2 min. Signal analyses were performed using the Fusion system (Peqlab, Erlangen, Germany).

### Results

**Fig. 1:**
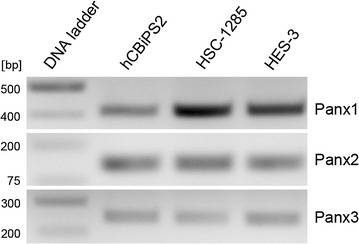
RT-PCR of Panx1, Panx2 and Panx3 in the investigated stem cell lines. All three pannexin mRNAs are expressed in all of the analyzed undifferentiated stem cell lines

**Fig. 2:**
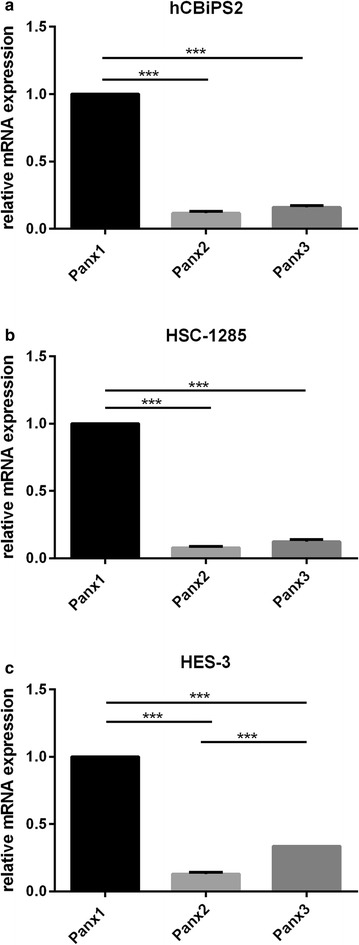
Relative expression of Panx1, Panx2 and Panx3 mRNAs in undifferentiated hCBiPS2, HSC-1285 and HES-3 stem cell lines, as determined using quantitative real time PCR. The mean Ct of the reference gene was subtracted from the mean Ct of the gene of interest (ΔCt). Expression was then normalized to Panx1 (relative expression = 1; ΔΔCt) Relative mRNA expression was calculated as 2^−ΔΔCt^ according to Pfaffl [25]. In comparison, Panx1 showed the highest expression in all of the stem cell lines: **a** in hCBiPS2 cells, Panx2 expression was 8.26-fold lower and Panx3 expression was 6.25-fold lower than Panx1 expression; **b** in HSC-1285 cells, Panx2 expression was 12.82-fold lower and Panx3 expression was 8.06-fold lower as compared to Panx1; **c** in HES-3 cells, Panx2 expression was 7.75-fold lower and Panx3 expression was 2.98-fold lower than Panx1 expression. ***p < 0.0001

**Fig. 3:**
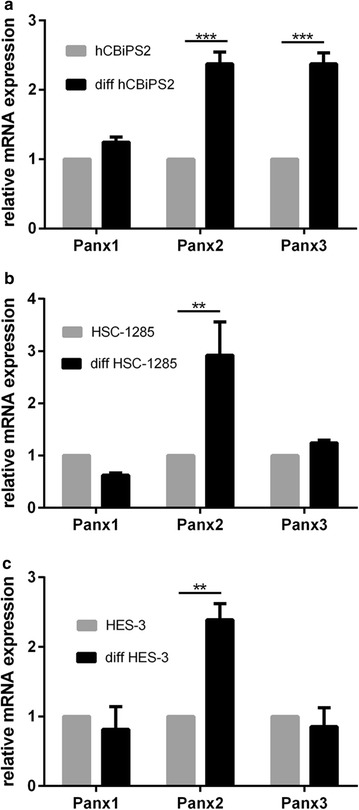
Relative gene expression of Panx1, Panx2 and Panx3 in undifferentiated and differentiated cells, as determined via quantitative real time PCR. The mean Ct of the reference gene was subtracted from the mean Ct of the gene of interest (ΔCt). Expression was then normalized to the values obtained for undifferentiated cells. Relative mRNA expression was calculated as 2^−ΔΔCt^ according to Pfaffl [25]. **a** In hCBiPS2 cells, relative expression of Panx1 was 1.25-fold higher after undirected differentiation, whereas the relative expression of Panx2 was 2.374-fold higher and Panx3 was 2.373-fold higher in differentiated cells compared to undifferentiated cells. **b** In HSC-1285 cells the relative expression of Panx1 was 1.6-fold lower after undirected differentiation, whereas Panx2 was 2.922-fold higher and Panx3 was 1.242-fold higher in differentiated cells compared to undifferentiated cells; **c** in HES-3 cells the relative gene expression of Panx1 was 1.22-fold lower after undirected differentiation, whereas Panx2 was 2.39-fold higher and Panx3 was 1.17 lower in undifferentiated cells compared to undifferentiated cells. In hCBiPS2 cells, HSC-1285 cells, and in HES-3 cells, expression of Panx2 was significantly higher in differentiated cells as compared to undifferentiated cells. In hCBiPS2 cells Panx3 expression was significantly higher in differentiated cells as compared to undifferentiated cells. **p < 0.001; ***p < 0.0001

In both undifferentiated induced pluripotent stem cells of the lines hCBiPS2 and HSC-1285 as well as in the undifferentiated cells of human embryonic stem cell line HES-3, Panx1-, Panx2- and Panx3-mRNAs were detected by RT-PCR (Fig. 1). Quantitative real time-PCR revealed that, among them, Panx1 showed clearly the highest expression (Fig. 2). The relative expression of Panx2 and Panx3 was approximately fivefold less than that of Panx1, and this was observed for all three cell types investigated in an undifferentiated state. In order to analyze the effect of an undirected differentiation on pannexin expression, all three cell lines were differentiated into embryoid bodies. To determine differential expression of germ-layer specific mRNAs, qPCR was performed on differentiated stem cells. As expected, each of the three stem cell lines expressed markers of all of the three germ layers upon undirected differentiation, i.e. the ectodermal marker WNT1 and Nestin, the endodermal marker TTR and AFP, as well as the mesodermal marker NKX2-5 and MYL7 (Additional file 2: Figure S1). Undirected differentiation of hCBiPS2, HSC-1285, or HES-3 stem cells hardly affected the expression of Panx1 as compared to its expression in the respective undifferentiated cells (Fig. 3). The expression of Panx2 was found significantly increased in all of the differentiated cell lines investigated (Fig. 3). Interestingly, Panx3 expression revealed a significant increase in hCBiPS2 cells only, whereas its expression in the other two cell lines remained unchanged (Fig. 3).

### Discussion

This is the first report on pannexin mRNA expression in human pluripotent stem cells. In three stem cells lines, including human induced pluripotent stem cells (hCBiPS2, HSC-1285) and human embryonic stem cells (HES-3), expression of Panx1, Panx2 and Panx3 was demonstrated. Upon undirected differentiation, which results in a population of cells representing all three germ layers, a differential regulation of mRNA expression was observed mainly for Panx2. To date, pannexin expression had been demonstrated in multipotent stem cells of various developmental lineages: Panx1 was shown to support proliferation of neural stem and progenitor cells via the release of ATP [20]. Panx3 is involved in the proliferation of osteo-progenitor cells [19]. Both Panx1 and Panx3 are involved in skeletal muscle myoblast proliferation and differentiation [10]. However, to our knowledge pannexin expression in human embryonic stem cells and induced pluripotent stem cells has not been analyzed yet. One major finding is that all three pannexin forms are expressed in the three investigated cell lines hCBiPS2, HSC-1285 and HES-3. Interestingly, the pattern of Panx1 to Panx3 mRNA expression was quite uniform between these cell lines with the relative expression of Panx1 exceeding that of Panx2 and Panx3 three- to fivefold. The higher expression of Panx1 might point to a general importance of this pannexin channel in pluripotent stem cells. In view of its ATP channel function, one might envisage the importance of an early ATP release and its tight regulation via Panx1. In particular, Panx2 shows a clear upregulation of gene expression in all investigated differentiated stem cell lines. Cx43 protein, previously shown to form ultra-structurally defined gap junction plaques in pluripotent stem cells [3], was detectable in lysates of undifferentiated as well as undirected differentiated cells. In contrast, the pannexin signals in the immunoblot were nearly absent (Additional file 3: Figure S2). These findings might reflect very low protein levels below the detection limit. However, they might also point to an intriguing situation: Panx1 and Panx2 proteins were previously detected in neural progenitor cells [20, 22]; Panx3 protein was demonstrated in osteoprogenitor cells [19]. Our study is the first one to investigate pluripotent stem cells and we did not detect Panx proteins. Despite the presence of pannexin mRNAs in pluripotent stem cells, protein translation might only occur in conjunction with a certain degree of differentiation towards multipotency.

### Limitations

The presented data are descriptive and the functional role of pannexins in stem cell-induced organogenesis remained unclear.

## Additional files

**Additional file 1.** Information on primers used for RT-PCR (**Table S1**) and for real time PCR (**Table S2**).

**Additional file 2: Figure S1.** Relative expression of germ layer-specific marker mRNAs in the differentiated stem cell lines (A) hCBiPS2, (B) HSC-1285, and (C) HES-3. The following symbols indicate a significant difference in relative mRNA expression with p < 0.05: # against each other marker; Δ against WNT1, TTR, AFP, and NKX2-5; □ against WNT1, TTR, NKX2-5, and o against WNT1, AFP, NKX2-5, and MYL7.

**Additional file 3: Figure S2.** Immunoblot against Cx43 and Panx1, Panx2 and Panx3 in undifferentiated (u) and differentiated (d) hCBiPS2, HSC-1285, and HES-3 cells. Protein extracts of cortex and Saos-2 cells served as positive controls for Panx1/Panx2 and for Panx3, respectively. Cx43 was detected in all cell lines, both differentiated and undifferentiated. The immune signals for Panx1, Panx2 and Panx3 were either negative or uncertain.

## Abbreviations

hCBiPS2: human cord-blood-derived induced pluripotent stem cell clone 2
hESC: human embryonic stem cells
HSC: hematopoietic stem cells
Panx: pannexin
