# Supplementary material for: Human stem cells express pannexins
Source: BMC Res Notes. 2018 Jan 22;11:54. doi: 10.1186/s13104-018-3125-z (PMC5778636; doi:10.1186/s13104-018-3125-z)
Supplement: Supplementary file 1 — Additional file 1. Information on primers used for RT-PCR (Table S1) and for real time PCR (Table S2). [file 13104_2018_3125_MOESM1_ESM.docx]

**Table S1: Primers for pannexins used in RT-PCR**

| **Primer** | **Sequence**  **5´-3´** | **Product size [bp]** | **Annealing T [°C]** | **Cycles** |
| --- | --- | --- | --- | --- |
| Panx1 fwd | GAGCTGGCTGTGGACAAGAT | 410 | 60 | 30 |
| Panx1 rev | TCTCTCATGTCAAGGTCACGC |  |  |  |
| Panx2 fwd | TCACCATCCTGAGCCGAAAC | 145 | 62 | 35 |
| Panx2 rev | ATGAGGATCTGCTGTGGGGC |  |  |  |
| Panx3 fwd | CATCTTCACCTCCGCCACTT | 247 | 60 | 35 |
| Panx3 rev | TTTGTCCCACCGACATAGCC |  |  |  |

**Table S2: Primers for pannexins and germ layer specific genes used in real time PCR**

| **primer** | **Sequence 5´-3´** | **Reference** |
| --- | --- | --- |
| 18S rRNA fwd | AAACGGCTACCACATCCAAG | (Beckmann et al., 2016) |
| 18S rRNA rev | CCTCCAATGGATCCTCGTTA | (Beckmann et al., 2016) |
| Panx1 fwd | AGAGCGAGTCTGGAAACC | (Ransford et al., 2009) |
| Panx1 rev | CAAGTCTGAGCAAATATGAGG | (Ransford et al., 2009) |
| Panx2 fwd | TCACCATCCTGAGCCGAAAC | n/a |
| Panx2 rev | ATGAGGATCTGCTGTGGGGC | n/a |
| Panx3 fwd | GGGACTCACTGCTTCACCAT | n/a |
| Panx3 rev | AACAGCAGATCGGAGCTGAG | n/a |
| TTR fwd (endodermal marker) | AAAACCAGTGAGTCTGGAGAGC | (Haase et al., 2009) |
| TTR rev (endodermal marker) | GTTGGCTGTGAATACCACCTCT | (Haase et al., 2009) |
| NKX2-5 fwd (mesodermal marker) | CCAGCCCTGCTCTCACG | (Haase et al., 2009) |
| NKX2-5 rev (mesodermal marker) | GCCCAGCGTAGGCCTCT | (Haase et al., 2009) |
| WNT1 fwd (ectodermal marker) | ACCTCTTCGGCAAGATCGTC | n/a |
| WNT1 rev (ectodermal marker) | CACACGTGCAGGATTCGATG | n/a |
| AFP fwd (endodermal marker | ATGAGCACTGTTGCAGAGGA | Primerdepot NIH |
| AFP rev (endodermal marker) | TACATTGACCACGTTCCAGC | Primerdepot NIH |
| MYL7 fwd (mesodermal marker) | TCAAGCAGCTTCTCCTGACC | Primerdepot NIH |
| MYL7 rev (mesodermal marker) | CTTGTAGTCGATGTTCCCCG | Primerdepot NIH |
| Nestin fwd (ectodermal marker) | GAGAGGGAGGACAAAGTC | G. Thiel, UdS |
| Nestin rev (ectodermal marker) | TCCCTCAGAGACTAGCGCAT | G. Thiel, UdS |

n/a not applicable. These primers were individually generated using the <http://www.ncbi.nlm.nih.gov/tools/primer-blast/> software.

Primerdepot NIH <https://primerdepot.nci.nih.gov/>
