# Supplementary figures and images for: Human stem cells express pannexins
Source: BMC Res Notes. 2018 Jan 22;11:54. doi: 10.1186/s13104-018-3125-z (PMC5778636; doi:10.1186/s13104-018-3125-z)

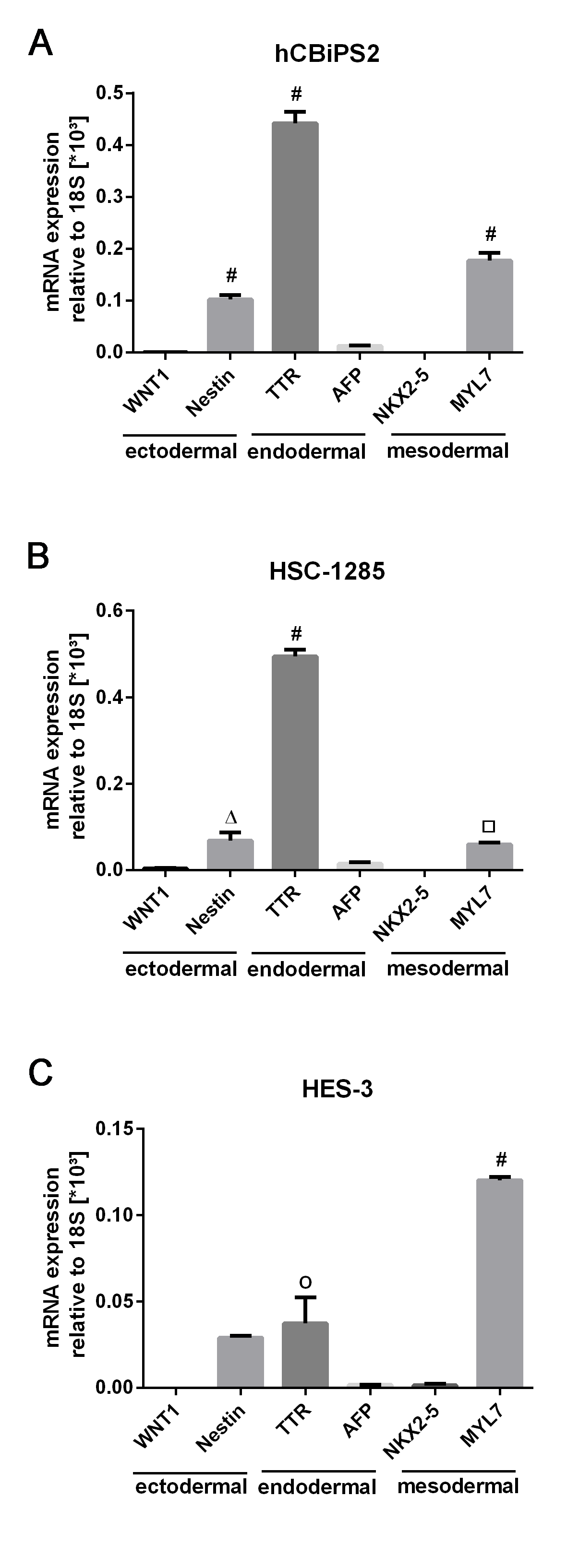

Supplement: Supplementary file 2 — Additional file 2: Figure S1. Relative expression of germ layer-specific marker mRNAs in the differentiated stem cell lines (A) hCBiPS2, (B) HSC-1285, and (C) HES-3. The following symbols indicate a significant difference in relative mRNA expression with p < 0.05: # against each other marker; Δ against WNT1, TTR, AFP, and NKX2-5; □ against WNT1, TTR, NKX2-5, and o against WNT1, AFP, NKX2-5, and MYL7. [file 13104_2018_3125_MOESM2_ESM.tif]

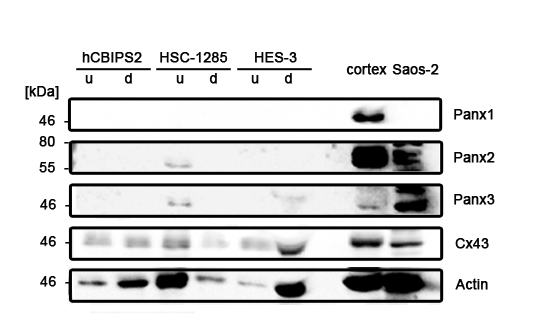

Supplement: Supplementary file 3 — Additional file 3: Figure S2. Immunoblot against Cx43 and Panx1, Panx2 and Panx3 in undifferentiated (u) and differentiated (d) hCBiPS2, HSC-1285, and HES-3 cells. Protein extracts of cortex and Saos-2 cells served as positive controls for Panx1/Panx2 and for Panx3, respectively. Cx43 was detected in all cell lines, both differentiated and undifferentiated. The immune signals for Panx1, Panx2 and Panx3 were either negative or uncertain. [file 13104_2018_3125_MOESM3_ESM.tif]
